# Intradermal injection of a Tat Oyi-based therapeutic HIV vaccine reduces of 1.5 log copies/mL the HIV RNA rebound median and no HIV DNA rebound following cART interruption in a phase I/II randomized controlled clinical trial

**DOI:** 10.1186/s12977-016-0251-3

**Published:** 2016-04-01

**Authors:** Erwann P. Loret, Albert Darque, Elisabeth Jouve, Elvenn A. Loret, Corinne Nicolino-Brunet, Sophie Morange, Elisabeth Castanier, Josiane Casanova, Christine Caloustian, Charléric Bornet, Julie Coussirou, Jihen Boussetta, Vincent Couallier, Olivier Blin, Bertrand Dussol, Isabelle Ravaux

**Affiliations:** ETRAV Laboratory, Faculty of Pharmacy, Centre National de la Recherche Scientifique (CNRS), Aix Marseille University, 27 Boulevard Jean Moulin, 13385 Marseille, France; Centre d’Investigation Clinique, Assistance Publique –Hôpitaux de Marseille (AP-HM), University Hospital Center (UHC) «la Conception», 147 Bd Baille, 13385 Marseille, France; Pharmacie Usage Interne, AP-HM, UHC «la Conception», 147 Bd Baille, 13385 Marseille, France; Centre de Pharmacologie Clinique et Evaluations Thérapeutiques (AP-HM), UHC «la Timone», 28 Boulevard Jean Moulin, 13385 Marseille, France; Unité Mixte de Recherche CNRS 5251, Institut de Mathématique de Bordeaux, CNRS, Bordeaux 2 University, 33000 Bordeaux, France

**Keywords:** HIV, Tat, Vaccine, Clinical trial, ART interruption

## Abstract

**Background:**

A Tat Oyi vaccine preparation was administered with informed consent to 48 long-term HIV-1 infected volunteers whose viral loads had been suppressed by antiretroviral therapy (cART). These volunteers were randomized in double-blind method into four groups (n = 12) that were injected intradermally with 0, 11, 33, or 99 µg of synthetic Tat Oyi proteins in buffer without adjuvant at times designated by month 0 (M0), M1 and M2, respectively. The volunteers then underwent a structured treatment interruption between M5 and M7.

**Results:**

The primary outcomes of this phase I/IIa clinical trial were the safety and lowering the extent of HIV RNA rebound after cART interruption. Only one undesirable event possibly due to vaccination was observed. The 33 µg dose was most effective at lowering the extent of HIV RNA and DNA rebound (Mann and Whitney test, p = 0.07 and p = 0.001). Immune responses against Tat were increased at M5 and this correlated with a low HIV RNA rebound at M6 (p = 0.01).

**Conclusion:**

This study suggests in vivo that extracellular Tat activates and protects HIV infected cells. The Tat Oyi vaccine in association with cART may provide an efficient means of controlling the HIV-infected cell reservoir.

## Background

The quest for an efficient vaccine against HIV has been a major issue since the initial cloning and identification of HIV-1 as the causative agent of AIDS [1, 2]. Although there is consensus that HIV eradication will require elimination of HIV infected cells that are mainly CD4 T lymphocytes (CD4), no cART and/or vaccine approaches have been able to reduce significantly the level of HIV infected cells in peripheral blood until now [3]. Different reasons to explain this lack of efficacy include the possibility that HIV variability is too high for the immune system [4]. The development of cART from 1996 showed that even with a low HIV-1 variability, the immune system is still unable to eradicate HIV infected cells [5]. The reservoir of HIV infected cells in the peripheral blood is made of cells at the latent state, which is remarkably stable since its eradication even with successful cART would require at least 70 years [6]. Why cytotoxic or CD8 T Lymphocytes (CTL) can not eliminate HIV infected cells remains controversial and it has been proposed that the central nervous system (CNS) could be a sanctuary (CTL have no access to CNS due to the blood–brain barrier) that would refresh peripheral blood with activated HIV infected cells [7]. However, the development of HIV DNA measurements points out CNS as a minor reservoir, while guts and rectal tissues called GALT and RALT are the major tissue reservoirs and CTL have access to theses tissues [8, 9]. Only GALT and RALT have HIV sequences compatible with the refreshment of peripheral blood with activated HIV infected cells [8]. Even patients with undetectable viraemia show evidence of ongoing viral replication in these tissue reservoirs and there is equilibrium with the peripheral blood compartment [10]. It was shown recently that HIV infected cells become rapidly resistant to CTL following HIV infection [3]. It could be possible that the capacity for HIV infected cells to survive in an environment containing CTL is due to the secretion from HIV infected cells of a HIV protein called Tat [11]. The presence of extracellular Tat in peripheral blood has been demonstrated in different clinical studies showing the presence of antibodies against Tat [11]. Extracellular Tat goes on to be secreted under ART and might protect HIV infected cells from CTL dues to its capacity to cross CTL membranes to trigger apoptosis [11]. Furthermore, extra cellular Tat might activate latent HIV infected cells and explain the rapid HIV rebound when cART is stopped [11]. However, there is a paucity of information on the role that extracellular Tat plays in infected subjects.

A single individual termed the “Berlin patient”, who was infected by HIV in 1995 is acknowledged to have been cured of HIV after having received a hematopoietic stem cell transplantation in 2007 from a compatible donor possessing the delta 32 mutation in CCR5 [12]. HIV DNA was no longer detectable in his peripheral blood in 2009 [13]. The complete eradication of HIV in this patient was demonstrated in 2012 by a retro seroconversion that is characterized by a significant decrease of antibodies against HIV [13]. A similar retro seroconversion was reported for the child termed the “Mississippi baby” at age of three [14]. HIV-1 antibodies were not detected in the child at 24, 26, and 28 months of age, but were found when she was 4 years old in 2014 due to a viral rebound [15]. Retro seroconversion has never been observed in patients receiving cART with no detectable viraemia and is therefore considered to be a marker for the elimination of HIV infected cell [11, 13]. In contrast, low-level viraemia that is below the limit of detection of viral RNA can be enough to sustain a high level of antibodies against HIV in spite of efficient ART [8].

Retro seroconversions were also observed after a survey of 2 years in 23 of 25 women who were seropositive in 1986 in Gabon but at the time this was interpreted as due to mutations in the HIV envelope protein [16]. A HIV strain termed HIV-1 Oyi was cloned from one of these women while she was still seropositive [16]. It was later shown that HIV Oyi had envelope proteins similar to other HIV strains and a correlation was established between the retro seroconvertion of these 23 women and the capacity for Tat Oyi to have immune properties never observed in other Tat variants [17]. This led to the testing of a Tat Oyi vaccine on rhesus macaques that were challenged by mucosal inoculation with a heterologous recombinant SHIV. The results showed that SHIV infected CD4 cells were no longer detectable in the peripheral blood 2 months after SHIV challenge in all macaques vaccinated with Tat Oyi and one of these animals retro seroconverted [18]. The efficacy of Tat Oyi was recently confirmed in another heterologous SHIV challenge [19]. It was also possible to identify a highly conserved surface on Tat variants and Tat Oyi has specific mutations that transform this highly conserved surface in a 3D épitope [20]. Tat Oyi can generate neutralizing antibodies against Tat variants whatever their mutations establishing a rational for testing Tat Oyi as active principle of a therapeutic vaccine in human trials [11].

This study describes the first clinical trial with Tat Oyi in a phase I/II double blinded and randomized controlled clinical trial. The primary outcome of phase I was the absence of serious adverse events (SAE) due to vaccination. The primary outcome for phase II was the capacity to control HIV RNA rebound in a vaccine group following treatment interruption. The secondary outcome for phase I and phase II was a specific immune response against Tat related to the vaccine and characterized by amplification and/or cross recognition of six Tat variants (including Tat Oyi) that are representative of the five main HIV-1 subtypes [11].

## Results

### Study design and eligibility criteria

The hypothesis was that neutralizing antibodies against Tat induced by this vaccine may help CTL to eliminate HIV infected cells. The Tat Oyi vaccine rationale is not to develop a Tat specific CTL response since HIV infected cells have GP120 at their surfaces and not Tat. Free extracellular Tat may constitute a screen that protects HIV infected cells from CTL. Neutralizing antibodies against Tat may destroy this screen. We proposed to test the Tat Oyi vaccine to HIV-1 infected volunteers who were virologically suppressed for at least 1 year while on ART. Three double blinded intradermal injections (500 µL) at Month 0 (M0), M1 and M2 of a solution having respectively 0, 11, 33 or 99 µg in four groups of volunteers. The vaccine was composed essentially of a synthetic Tat Oyi in 100 mM Phosphate buffer pH 4.5 and 9 g/L NaCl. Three intradermal injections were carried out in each group and the only difference with the placebo group was the absence of Tat Oyi. No adjuvant or other compounds were added. Volunteers agreed to stop cART for 2 months between M5 and M7, but cART could be resumed if a volunteer was >100 copies/mL HIV RNA at M6. Observance of cART and cART interruption between M5 and M7 were monitored by detection of antiretroviral levels in blood samples over the course of the study.

Volunteers had been recruited from among long term suppressed HIV infected patients who did not experience cART in early infection. cART interruption of 2 months is considered to be safe for these HIV infected patients with a CD4 counts >350 cells/mm^3^ and in whom a limited decrease of about 65 CD4 cells/mm^3^ has been observed [21, 22]. HIV RNA rebound, following cART interruption for these patients is characterized by the appearance of detectable HIV RNA at day 15 that increases until stabilization (up to 30 days) with a mean 10,000 (log 4) HIV RNA copies/mL [21]. This dynamic can be different for HIV patients who received cART during primary infection [15, 23, 24], perhaps because early initiation of cART may reduce the size of the HIV DNA reservoir [<1.5 log HIV DNA copies per 10^6^ peripheral blood mononuclear cells (PBMC)] potentially influencing HIV RNA rebound when cART is interrupted [23]. Levels of HIV DNA in peripheral blood of patients receiving cART (without early initiation) since <10 years appear to be constant with a median of 1.9 log HIV DNA copies per 10^6^ PBMC and a mean difference of 0.2 log copies per 10 years of effective cART [25, 26].

### Base line and safety

**Fig. 1:**
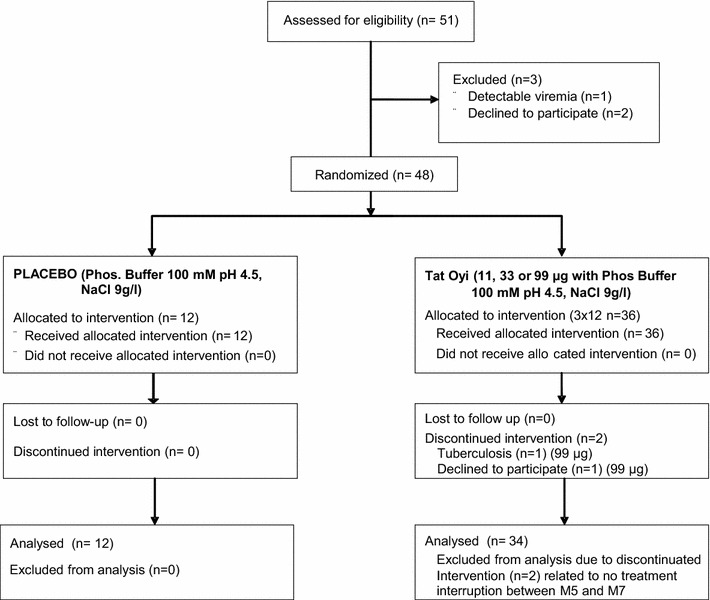
EVATAT flow diagram. This flow diagram was constructed according to CONSORT recommendation [41]

Fifty-one volunteers were initially included and filled a written informed consent but only 46 followed the protocol to the end. The mean and median age was 46 years old, 27 % (n = 13) were women, they had no co infection with hepatitis viruses, they have CD4 >350 cells/mm^3^ and were never under 200 CD4 cells/mm^3^ since 12 months (see Table 1 for base line and EVATAT in clinicaltrial.gov for the complete inclusion and exclusion criteria). Only one SAE (a *facial neuralgia* for 1 week) that occurred 11 months after vaccination with three 11 µg vaccine doses was considered as possibly related to vaccination (Table 2). The participant flow diagram shows that four other patients had a Serious SAE between first assessment in March 2013 and when the last patient left the trial at M12 in December 2014 (Fig. 1). One of these four SAE occurred between the time of assessment and the first vaccination (M0) and another SAE was a chirurgical intervention programmed before vaccination (Table 2). The two other SAE were tuberculosis and a diarrhea related to ART and it is not possible to establish a relationship with vaccination.

**Table 1:** Base line

*Volunteers characteristics*	
Variable	Total (n = 46)
Age (years)	
Mean	46.4 (±9.7)
Median (Q1–Q3)	47.0 [42.0–51.5]
Sexe n (%) woman	13 (27 %)
HIV infection diagnosis (years)	
Mean	12.4 (±7.9)
Median (Q1–Q3)	12.0 [4.8–20.3]
Years since HIV RNA <40 copies/mL	
Mean	6.0 (±4.3)
Median (Q1–Q3)	4.6 [2.1–9.7]
ART	
Nucleoside base	1 (2.1 %)
Non nucleosidic	28 (58.3 %)
Protease	15 (31.3 %)
Integrase	4 (8.3 %)
HIV DNA (copies/10^6^ PBMC)	
Mean	86.7 (±117.2)
Median (Q1–Q3)	49.5 [1.0–111.0]
Log HIV DNA	
Mean	1.7 (±0.5)
Median (Q1–Q3)	1.7 [0.0–2.1]
CD4 (cells per µL)	
Mean	692.4 (±259.6)
Median	666.0 [515.0–791.5]
CD8 (cells per µL)	
Mean	688.3 (±309.8)
Median (Q1–Q3)	654.0 [475.0–753.0]
CD4/CD8	
Mean	1.1 (±0.4)
Median (Q1–Q3)	1.1 [0.8–1.4]
Nadir (CD4 <350 cells per µL) (years)	
Mean	5.8 (±4.9)
Median (Q1–Q3)	4.4 [2.0–8.3]
Nadir CD4 (cells per µL)	
Mean	336.3 (±114.0)
Median (Q1–Q3)	312.0 [265.0–390.0]

**Table 2:** Serious adverse events (SAE) (phase I primary end point)

Volunteers	Groups (µg)	Events	Time^a^	Due to vaccine
No 2	11	Facial neuralgia	M11	Possible
No 24	99	Diarrhea	M3	Doubtful
No 36	99	Tuberculosis	M3	Doubtful
No 41	33	Hernia hiatus repair	M9	Not possible
No 47	33	Haemorrhoids	D-7	Not possible

^a^Time is expressed in months (M) after the first injection or in days (D) before the first injection

### HIV RNA

**Fig. 2:**
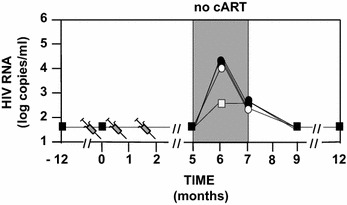
Design of the study and HIV RNA Rebound. HIV-1 infected volunteers (n = 46) were randomized into four groups having at month 0 (M0), M1 and M2 double blinded intradermal injections with respectively 0 (n = 12-*black square*), 11 µg (n = 12-*black circle*), 33 µg (n = 12-*white square*) or 99 µg (n = 10-*white circle*) of a synthetic protein called Tat Oyi in a saline buffer without adjuvant. The volunteers stopped their antiviral treatment between M5 and M7. Volunteers had undetectable HIV RNA since at least 1 year before vaccination and treatment interruption. HIV RNA rebound is displayed as the median for each group on a logarithm scale. Four volunteers did not resume ART at M6 in the 33 µg group while only two volunteers resumed ART in the other groups. HIVRNA at M7 is depicted for volunteers who resumed ART at M6 (n = 36) and at M7 (n = 10)

**Fig. 3:**
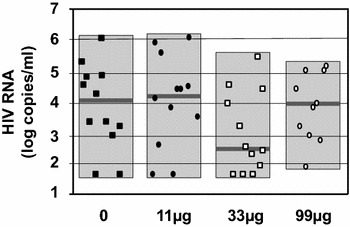
HIV RNA at M6 for each volunteer. The HIV RNA level (copies/mL) is displayed on a logarithmic scale. For each group, the median is shown as a *grey line*

The 46 volunteers did not have detectable HIV RNA for at least 1 year before to stop cART at M5 (Fig. 2). Ten volunteers did not resume cART at M6. The primary outcome of the phase IIa study was reached with 33 µg showing a lowering of the extent of HIV RNA rebound median from 4.1 to 2.6 log copies/mL (Fig. 2) with eight volunteers (66 %) being <3.5 log copies/mL (Fig. 3). Four volunteers (33 %) in the 33 µg group were able to maintain their viremia <2 log copies/mL after cessation of cART (Fig. 3) and did not resume their treatment at M6. In the three other groups (including the placebo group) two volunteers did not resume cART at M6. We observe in Table 3 that the analysis of the four groups confirmed that 33 µg gave the best control of RNA rebounds with the lowest median. A one sided Mann and Whitney test that compared the placebo and the 33 µg group showed that differences between the two were borderline significant (p = 0.07), while the two other vaccine groups yielded none significant differences (p > 0.1).

**Table 3:** Vaccine efficacy to prevent HIV RNA rebound (phase II primary end point)

Tat Oyi	0 (n = 12)	11 µg (n = 12)	33 µg (n = 12)	99 µg (n = 10)
Medians (copies/ml)	12,921	20,706	442	9475
Total range	39–0.9 × 10^6^	39–1.2 × 10^6^	39–0.4 × 10^6^	81–0.1 × 10^6^
Log medians	4.1	4.3	2.6	4.0
*p* values*		0.38	0.07	0.50

HIV RNA at M6 after cART interruption at M5

* *p* values were determined for each vaccine group versus placebo with a one sided Mann and Whitney test without adjustment. Undetectable viraemia (<40 copies/ml) were replaced by 39 (1.6 log) copies/ml

### CD4 and CD8 cell

**Fig. 4:**
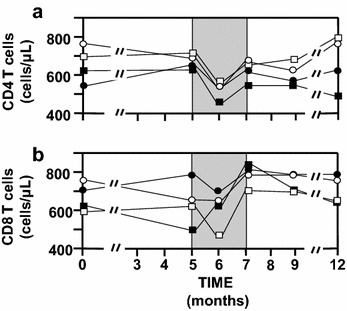
Levels of CD4 and CD8 lymphocytes. **a** Median CD4 count for each group. **b** Median CD8 count for each group. Group 1 is depicted as a *black square*, group 2 as a *black circle*, group 3 as a *white square* and group 4 as a *white circle*

The 11 µg group seemed to yield a significant increase (variation >65 CD4 cells/mm^3^) from 554 (total range or TR was 418–1067) to 636 (TR 502–1124) CD4 cells/mm^3^ at M5 (Fig. 4a). Mann and Whitney test (M5 vs. M0) confirmed that 11 µg had a significant increase (p = 0.001) compared to 33 µg (p = 0.22) and 99 µg (p = 0.92) in a Mann and Whitney test. After treatment interruption, the four groups had a similar decrease from 671 (TR 533–773) to 519 (TR 453–677) CD4 cells/mm^3^ for n = 46. No effect of the vaccine was detectable on CD4 levels following treatment interruption. However, the 33 µg group at M12 had an increase compared to M0 from 706 (TR 542–783) to 784 (TR 554–837) CD4 cells/mm^3^, while the placebo group had a decrease between M0 and M12 going from 615 (TR 436–781) to 476 (TR 436–677) CD4 cells/mm^3^ (Fig. 4a). Higher variability was observed with CD8 levels, especially for the placebo group CD8 level with the 11 µg group being significantly higher at M5 (p = 0.004). The CD8 level with the 33 and 99 µg groups appeared to be higher after treatment interruption (Fig. 4b). The median of the CD4/CD8 ratio was <1 for the 12 months of the study excepted at M6, where it was 1.25 with 33 µg and 0.96 with 99 µg, while it was 0.75 with the placebo and 0.88 with 11 µg.

### HIV DNA

**Fig. 5:**
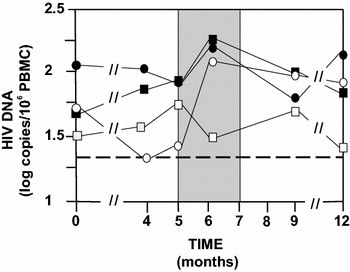
HIV DNA in peripheral blood. HIV DNA medians for each group are shown from M0 to M12. Volunteers with undetectable HIV DNA (<20 copies/10^6^ PBMC) were counted as having respectively 19 copies, which is 1.3 log copies/ml (*dashed line*). The placebo group is depicted as a *black square*, the 11 µg group as a *black circle*, the 33 µg group as a *white square* and the 99 µg group as a *white circle*. Median decreases in HIVDNA levels were observed at doses of 99 µg (n = 10) from 1.8 (TR 1.3–2.5) to 1.3 (TR 1.3–2.6) log copies HIV DNA/10^6^ PBMC between M0 and M4 and is due to six volunteers with HIV DNA copies <20. The absence of HIVDNA rebound at M6 with the 33 µg group is highly significant in a Mann and Whitney test (*p* = 0.001). At M12, the 33 µg group has a median of 1.4 log copies/ml that is very close of the undetectable level. However, this difference is not significant (*p* > 0.1) compared to M0

Cell-associated total HIV DNA measured in this study can not be considered as a true measurement of HIV-1-infected cells that persist in peripheral blood during cART, which are the source of rebound viremia upon stopping therapy. This HIV DNA measurement count both integrated HIV DNA in HIV infected cells and free HIV DNA resulting from HIV infected cells desegregation. The most significant decrease in HIV DNA was observed with the 99 µg group (n = 10) between M0 and M4 (Fig. 5). Median decreases from 1.8 (TR 1.3–2.5) to 1.3 (TR 1.3–2.6) log copies HIV DNA/10^6^ PBMC were observed in six volunteers having HIV DNA <20 copies at M4. However, an equivalent increase occurred between M4 and M5 (before treatment interruption) and continued after treatment interruption at M6. Furthermore, the difference between M0 and M4 with 99 µg is not significant (p = 0.27). Only the 33 µg group did not show a HIV DNA rebound after treatment interruption at M6 with five volunteers having HIV DNA <20 copies. The absence of HIV DNA rebound is significant with a Mann and Whitney test (p = 0.001) compared to 99 µg (p = 0.1) and 11 µg (p = 0.49). The HIV DNA decrease with 33 µg was less spectacular but was prolonged from M0 to M12 (Fig. 5). At M12, the 33 µg group has a median of 1.4 log copies/mL that is very close of the undetectable level, but this difference is not significant (p > 0.1) compared to M0. Four volunteers with 33 µg and three volunteers with 99 µg were still having HIV DNA <20 copies at M12. This result suggests that extracellular Tat probably protects latent HIV infected cells.

### IgG anti Tat response

**Fig. 6:**
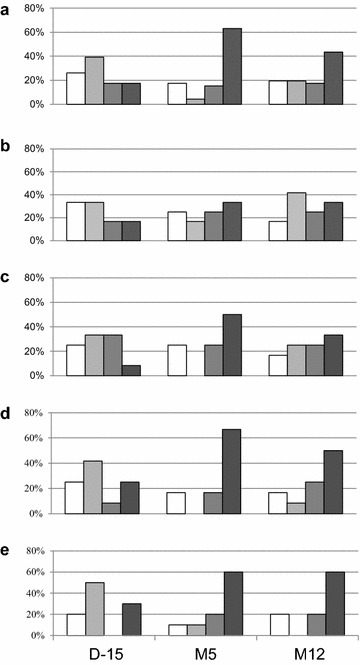
Evolution of the IgG anti Tat immune response in ELISA test. Data are shown before vaccination (D-15), after vaccination at M5 (just before ART interruption) and at M12 (5 months after ART was resumed). The volunteers were classified in four categories: The *white bar* corresponds to no recognition of Tat variants. The *different shades of grey* correspond to the capacity for volunteers to recognize one (low responder), two (moderate responder) or three to six (high responder) Tat variants. **a** Evolution of the Tat immune response for all volunteers (n = 46). **b** Evolution of the Tat immune response in the placebo group (n = 12). **c** Evolution of the Tat immune response in the 11 µg group (n = 12). **d** Evolution of the Tat immune response in the 33 µg group (n = 12). **e** Evolution of the Tat immune response in the 99 µg group (n = 10)

Immune responses against Tat were based on the capacity to recognize and/or increase recognition of six different Tat variants representative of the five main HIV-1 subtypes in a sandwich ELISA test [11]. Four categories were observed with recognition of none, recognition of one Tat variant (low responder), recognition of two Tat variants (moderate responder), and recognition of three to six Tat variants (high responder). Prior to vaccination, specific responses against Tat were mostly low (39 %) or non-existent (26 %) (Fig. 6). Only 17 % were high responder but this increased to 63 % (n = 29) at M5 after vaccination. Five volunteers who had no Tat response before vaccination never developed an immune response against Tat in spite of three injections of either 11 µg (n = 2), 33 µg (n = 2) and 99 µg (n = 1). Vaccination in two volunteers had an immune response against Tat unchanged before and after vaccination, one who receive 11 µg while the other received the 99 µg dose. Therefore, 7/34 volunteers (20 %) did not respond to Tat Oyi injection. Four volunteers who where high responders prior to vaccination did not display responsiveness after injection of 33 µg (n = 1) and 99 µg (n = 3) of Tat Oyi. The highest increase in Tat immune response at M5 was seen with the 33 µg dose since eight patients (67 %) have a high immune response against Tat (Fig. 6d). To try to determine the role of anti Tat immune responsiveness in HIV RNA rebound, we compared at M5 the high responders (n = 28) with the three other groups (n = 18). The high responders median was 3.3 while the median for the three other groups was 4.6 log copies/mL (p = 0.01). This finding suggests that the anti Tat immune response may prevent the ability of extracellular Tat to activate latently HIV infected cells.

## Discussion

The only successful phase II vaccine clinical trial against HIV used a recombinant virus triggering envelope proteins (RV144), and demonstrated an efficacy of about 30 % at preventing HIV infection [27]. Two previous phase II clinical trials were performed with Tat fragments (TUTI-16) [28] or with recombinant Tat (ISST-02) [29]. Interestingly, in these two clinical trials as in this one, a dose of 30 µg of Tat yielded the best results [28, 29]. Three years after vaccination in the ISST-02 protocol, a significant increase in CD4 cells and a HIV DNA decrease of 0.2 log copies/10^6^ PBMC occurred but there was no placebo group to determine if these results were related to the vaccine [30]. Another phase II clinical trial with the same vaccine (ISST-03) is ongoing in South Africa [30].

The Tat Oyi vaccine is entirely synthetic and no adjuvant or compounds such as interleukins were added to amplify the immune response. The results presented in this study are therefore due only to Tat Oyi, and the differences observed between the three different doses employed and the placebo are related only to the quantity of Tat Oyi injected. HIV RNA rebound following treatment interruption was chosen as a primary outcome to evaluate vaccine efficacy in phase II (M5 to M12). Tat Oyi is the first vaccine to demonstrate efficacy in the context of cART interruption, even though 21 other trials have tried and failed to show differences from the placebo group [31]. It is important to outline that lowering the extent of HIVRNA rebound to <0.5 log copies is considered as significant [31]. Since we have 8 volunteers <3.5 log copies/mL in the 33 µg group, it suggests an efficacy of 66 % for the Tat Oyi vaccine regarding the HIV RNA rebound. The immune response against Tat was monitored by ELISA test using Tat variants representative of the five main HIV-1 subtypes [11]. Previously, we showed using this ELISA test that an immune response against Tat variants was present for at least 50 % of HIV infected patients in African and European cohorts due to endogenous extracellular Tat [11]. Furthermore, we showed that this anti-Tat immune response could often be transient [11]. This study shows that anti-Tat immunity can help to control HIV RNA rebound and that the anti-Tat Oyi response may help to lower HIV DNA.

This study confirms in humans the important role of extracellular Tat in activating latent cells. Latency is a way for HIV, as other defective viruses, to maximize gene expression while avoiding global T cell activation that would involve the rapid elimination of HIV infected cells since activated T cells can survive for only a few days [4]. Different molecular mechanisms of HIV latency have been recently reviewed including Tat and other transcription factors such as NF-κB, the cyclin dependent kinases CDK13 and CDK11, and the positive transcription elongation factor b (P-TEFb) [32]. It is interesting that different strategies called “shock and kill” have been proposed to cure AIDS with agents that activate these proteins or release P-TEFb [32]. An anti-Tat compound such as Dihydro-cortistatin A can suppress HIV transcription but also inhibits HIV-1 mediated neuro inflammation [33]. The blood–brain barrier appears to be damaged in HIV infected patients, and extracellular Tat may be involved in this process [33]. This damage may be due to CTLs that are present in the CNS in the mouse model [34]. This recent discovery does not fit well with the role of sanctuary that the CNS is thought by some to play for HIV infected cells. The Tat Oyi vaccine may help to diminish HIV-associated neurocognitive disorders.

## Conclusion

The intradermal administration of the Tat Oyi vaccine was safe and could attain the effects described without adjuvant. The lack of efficacy of the vaccine at a dose of 99 µg from M5 may be due to a collapse of the Tat immune response before M5. Tat Oyi is the first therapeutic vaccine to show a measure of success in regard to both HIV RNA and DNA in a phase II clinical trial. The Tat Oyi vaccine appears to reduce HIV DNA and potentially the numbers of HIV infected cells in peripheral blood. Preliminary results show that three volunteers who received 33 or 99 µg are still HIV DNA undetectable at M24. The Tat Oyi vaccine together with cART may provide a means of control of HIV infection.

## Methods

### Participants

The protocol was entitled: “Evaluation in seropositive patients of a synthetic vaccine targeting the HIV Tat protein” with the acronym: “EVATAT”. The protocol was registered in the European Clinical trial data base (EudraCT) with the ID number: 2012-000374-36. The protocol got a favorable advice from an ethic committee (CPP SudMed 2) on November 9th, 2012 and an authorization from the French drug agency (ANSM) on January 14th, 2013 and was registered in *clinicaltrial.gov* with the reference NCT01793818. A written informed consent was obtained from all participants. This was a monocentric clinical trial located in the University Hospital Center “la Conception” in Marseille (Provence). All the volunteers were French citizens living in different cities in Provence, the average age was 46 years old with the oldest being 64 years old and the youngest 32 years old. The main inclusion criteria were: Age from 18 to 64 years old, on effective ART for at least 12 months with a viral load <40 copies/mL. The main exclusion criteria: Patients in HIV-1 primo infection, HIV-2 infected, having antibodies against HBV, HCV or HTLV-1 viruses, chronic active infections, immunosuppressive therapy, cancer, pregnancy and breastfeeding. Adverse events were monitored and clinical examination performed at each visit, with blood sampling for safety (general biochemistry and hematology). HIV RNA was analyzed by conventional methods with respectively a cut off of 40 copies per mL and HIV DNA HIV-1 DNA levels were measured by using the Generic HIV^®^ assay (Biocentric, Bandol, Provence) with a cut off of 20 copies/million of PBMC. CD4+ and CD8+ T cell counts were analyzed by conventional methods.

### Vaccine preparation

The active principle is a synthetic protein of 101 amino acid residues termed Tat Oyi [11]. Tat Oyi was synthesized in the ETRAV laboratory (Faculty of Pharmacy, Marseille, Provence) in Fmoc solid phase synthesis with a synthesizer APPLIED 433A in one run as previously described [35]. Two clinical lots (Freeze dried 33 µg Tat Oyi and Phosphate buffer 100 mM pH 4.5 NaCl 9 g/L) were produced by the pharmaceutical company ELIAPHARM at Sofia Antipolis (Provence). The two clinical lots were sterile and stable for 3 years at 4 °C. The vaccine was reconstituted by the CHU Conception hospital pharmacy on the day of administration by mixing freeze dried Tat Oyi (0, 11, 33 or 99 µg) with the buffer.

### Immune response against Tat

The antibody response against Tat was monitored with an ELISA test using Tat variants representative of the five main HIV-1 subtypes and Tat Oyi [11] in the ETRAV laboratory. For each volunteer, six blood samplings were carried out at M0, M2, M5, M7, M9 and M12 and sera were extracted and stored at—25 °C. For each patient and for each blood sampling the presence of IgG against six Tat variants was tested on six sera dilutions (1/3, 1/9, 1/27, 1/81, 1/243, 1/729, 1/2181, 1/6561). The ELISA tests were made in 2 days and the first day the six Tat variants were coated on 96 wheels NUNC plates in duplicate. We had six sera from each patient at M0, M2, M5, M7, M9 & M12 and one plate per serum was used. The second day, the six sera of a same volunteer were added with the same dilutions (one sera for one plate) and the association Tat variants/Human IgG in the six dilutions was revealed with peroxide conjugate Goat anti Human IgG and the colorimetric ABTS reaction at 433 nm, measured with a BIOTEK spectrophotometer. The six plates were measured the same day with a delay of 45 min corresponding to the time of incubation for each plate with ABTS. The intra assay variability of the ELISA test was monitored at the end of the second day with OD values that had to have a standard deviation <0.2 OD between a same Tat variant and a same dilution in the duplicate of a same plate. Inter assay variability was monitored with ELISA test that had to be reproducible at least twice for each volunteer with a delay of 6 months between two experiments. After vaccination, a specific response due to the vaccine was characterized by the apparition of an immune response against Tat variants for volunteers who had no response before vaccination. For volunteers who had an immune response against Tat before vaccination, a specific response due to the vaccine was characterized by recognition at a lower dilution of the same Tat variant and/or recognition of new Tat variants.

### Randomization and masking

It was a mono centric clinical study and it was double-blind with respect to vaccine dose assignment. The injections occurred in the “Centre d’Investigation Clinique” (CIC) in the UCH “la Conception”. The volunteers and the investigators in the CIC were unaware of the content in the syringes. Pharmacists, from the “Pharmacie à Usage Interne” (PUI) in the UCH “la Conception”, prepared and distributed the syringe the morning of the arrival of each volunteer in the CIC according to a schedule previously planned with the volunteer and in agreement with the EVATAT protocol. Volunteers (n = 48) were randomly assigned (1:1:1:1) to receive either 0, 11, 33 or 99 µg of a synthetic Tat Oyi complemented with a saline buffer. Randomization was carried with volunteers stratified in four block size named A, B, C and D and the vaccine doses corresponding to each letter was not known. The randomization scheme was prepared by Dr. Albert Darque in the PUI and securely stored with restricted access. No request to unmask study participants was asked for EVATAT study and the randomization was unmasked only on September 15th, 2015 after the “freezing” of EVATAT data from all investigators.

### Statistical analysis

The sample sizes have not been evaluated with a statistical methodology including power analysis. Nonparametric testing procedures were planned, and multifactorial analysis of RNA, DNA, CD4, CD8 was the core of the study. It was not possible to use standard tools to determine sample sizes according to a pre-specified power of the analysis (that requires a given value for the detectable difference). A total amount of 48 participants divided in four groups was accepted by authorities for a phase I/IIa clinical trial, and ensured enough sample sizes for an exploratory statistical analysis. Volunteers with undetectable HIV RNA (<40 copies/mL) or HIV DNA (<20 copies/10^6^ PBMC) were counted according to the “worst case scenario”, which were 39 copies for HIV RNA and 19 copies for HIV DNA (respectively 1.6 log and 1.3 log. The non parametric Mann and Whitney test [36] was used as a standard test. All p values are one-sided because of an a priori knowledge of the expected variation between placebo and vaccine groups. To compare vaccinated groups with a placebo require multiple comparisons that may induce type I family wise errors when non adjusted p values are used to detect significant differences between groups [37–39]. This is especially the case for large number of groups and unforeseen comparisons. Our analyses do not suffer from this problem because only three groups are compared to the placebo. Moreover, these comparisons were planned in the statistical analysis plan of the study and have not to be considered as unplanned subgroup comparisons. Thus, despite the fact that the sample sizes was most of the time not sufficient enough to ensure a statistical significance of the observed differences by adjusting for multiples comparisons, we provided non adjusted p values for one-sided Mann and Whitney tests as elements for discussion. An overall Kruskal–Wallis test would not have revealed a statistically significant difference among groups, mainly due to a lack of power in comparison of one control group versus the three other groups with 48 patients. The Consort 2010 [40] warns against the risk of spurious findings, that is why these statistical data must be seen as an exploratory analysis.
