# Erratum to: Intradermal injection of a Tat Oyi-based therapeutic HIV vaccine reduces of 1.5 log copies/mL the HIV RNA rebound median and no HIV DNA rebound following cART interruption in a phase I/II randomized controlled clinical trial

**DOI:** 10.1186/s12977-016-0264-y

**Published:** 2016-05-09

**Authors:** Erwann P. Loret, Albert Darque, Elisabeth Jouve, Elvenn A. Loret, Corinne Nicolino-Brunet, Sophie Morange, Elisabeth Castanier, Josiane Casanova, Christine Caloustian, Charléric Bornet, Julie Coussirou, Jihen Boussetta, Vincent Couallier, Olivier Blin, Bertrand Dussol, Isabelle Ravaux

**Affiliations:** ETRAV Laboratory, Faculty of Pharmacy, Centre National de la Recherche Scientifique (CNRS), Aix Marseille University, 27 Boulevard Jean Moulin, 13385 Marseille, France; Centre d’Investigation Clinique, Assistance Publique –Hôpitaux de Marseille (AP-HM), University Hospital Center (UHC) «la Conception», 147 Bd Baille, 13385 Marseille, France; Pharmacie Usage Interne, AP-HM, UHC «la Conception», 147 Bd Baille, 13385 Marseille, France; Centre de Pharmacologie Clinique et Evaluations Thérapeutiques (AP-HM), UHC «la Timone», 28 Boulevard Jean Moulin, 13385 Marseille, France; Unité Mixte de Recherche CNRS 5251, Institut de Mathématique de Bordeaux, CNRS, Bordeaux 2 University, 33000 Bordeaux, France

## Erratum to: Retrovirology (2016) 13:21 DOI 10.1186/s12977-016-0251-3

The original version of this article unfortunately contained a mistake. The “Acknowledgement” section was incorrect and included Dr. Catherine Tamalet. Dr. Catherine Tamalet should not be included in the “Acknowledgement” section for the following reasons:

Dr. Catherine Tamalet requests to be withdrawn from the “Acknowledgements” section in this article, on the following grounds: (a) No permission was asked of her to be acknowledged. (b) She did not read the manuscript before its publication. (c) The method depicted in the “Methods” section to quantify HIV DNA [“Generic HIV assay (Biocentric, Bandol, Provence) with a cut off of 20 copies/million of peripheral blood mononuclear cells (PBMC)”] is not the method employed in her laboratory. Dr. Tamaret routinely employs an “in-House” method with a cut-off of 20 copies. Dr. Thamalet was in the acknowledgement for fruitful discussion and not for her participation to the study.

Dr. Catherine Tamalet has been removed from the original article [1].
